# A fragment-based electrophile-first approach to target histidine with aryl-fluorosulfates: application to hMcl-1

**DOI:** 10.21203/rs.3.rs-6214862/v1

**Published:** 2025-03-26

**Authors:** Giulia Alboreggia, Kendall Muzzarelli, Zahra Assar, Maurizio Pellecchia

**Affiliations:** 1 Division of Biomedical Sciences, School of Medicine, University of California Riverside, 900 University Avenue, Riverside, CA 92521, USA.; 2 Cayman Chemical Co., 1180 E. Ellsworth Road, Ann Arbor, MI 48108.

**Keywords:** Histidine covalent, aryl-fluorosulfates, fragment-based drug design, hMcl-1, electrophile-first, drug discovery, fragment-based drug design, fragment-based ligand design

## Abstract

Aryl-fluorosulfates are mild electrophiles that are very stable in biological media and *in vivo* and can efficiently react with the side chains of Lys, Tyr, or His residues, when properly juxtaposed by a high-affinity ligand. A more powerful approach to derive novel ligands would consist in starting from the covalent adduct and building the ligand off those initial interactions. While this strategy has been proven for Cys with molecular fragments containing Cys targeting electrophiles such as acrylamides, a corresponding strategy with fluorosulfates targeting His/Lys/Tyr residues has yet to be reported. We report here that a fragment library of aryl-fluorosulfates, when deployed with proper biophysical screening strategies, can identify initial covalent fragments. We report on novel strategies to enhance the success rate of such *electrophile-based fragment screening* for His/Lys/Tyr residues and to characterize the resulting hits. As an application we report on novel covalent fragment hits targeting hMcl-1 His 224.

## Introduction

The design of irreversible covalent drugs has proven beneficial in drug discovery in the past decade, resulting in the approval of several Cys-covalent therapeutics, especially in oncology^1–7^. The increased pharmacodynamics of covalent ligands makes this approach particularly attractive to increase the binding affinity of ligands targeting protein-protein-interactions (PPIs) for which it has been notoriously difficult to attain greater potency^8–10^. Moreover, covalent drugs present also pharmacokinetics advantages *in vivo* compared to reversible drugs, providing a more sustained inhibition of the target. Acrylamide-based Cys-covalent inhibitors Osimertinib (EGRF, NSCLC)^11^, Ibrutinib (BTK, CLL)^12^, Neratinib^13^ and Afatinib^14^ (HER2 and EGFR, various solid tumors), have been all approved by the FDA in very recent years. Moreover, FDA approved peptide-like irreversible agents Bortezomib (Velcade) and more recently Carfilzomib (Kyprolis)^15^, that are Ser-covalent proteasome inhibitors based on boronic acid and epoxide, respectively. Very recently Sotorasib (Lumakras) was the first KRAS mutant inhibitor approved by the FDA, targeting covalently the KRAS(G12C) mutant^16–19^. The latter was discovered using an “electrophile-first” approach that identified for the first time a targetable pocket on the surface of KRAS^16–19^. However, Cys residues are rarely found at protein interfaces and, with only a few exceptions with peptide ligands,^20,21^ small molecules targeting PPIs based on Cys-covalent agents remain scarce. Hence, recently several new electrophiles have been proposed to target other residues such as lysine (Lys), histidine (His), tyrosine (Tyr)^3–5,22–32^, and more recently also other residues such as arginine (Arg)^33^, and aspartic acid (Asp)^34^. Among those, we and others reported that certain aryl-sulfonyl fluorides and aryl-fluorosulfates can react to His^24,29,35,36^, Lys^29^, or Tyr^3–5,22–32^ when properly incorporated in carrying ligands to be juxtaposed to a targeted residue. We also recently found that targeting His residues is particularly attractive given the favorable nucleophilicity of His and the frequency of histidine residues in binding sites^22,37^. We recently also reported that certain stabilized sulfonyl fluorides can be used to target Lys or His residues^25,27,29^ and other reports suggest that certain sulfonyl triazoles^38,39^ or even chloro-acetamides can also be deployed to target His residues^40^. However, and in view of their anticipated use as pharmacological tools or therapeutics, our studies focused on the less reactive aryl-fluorosulfates. We recently demonstrated that when properly incorporated in existing binding ligands (hence using a “ligand-first” approach), this milder electrophile can result in potent Lys or His covalent agents that are also orally bioavailable in mice^1,4,5,41,42^. The question then arises on whether aryl-fluorosulfates can be also used generally to derive novel covalent ligands targeting His, Lys, or Tyr residues, using an “electrophile-first” strategy, whereby a fragment-library of aryl-fluorosulfates is assembled and screened by biophysical methods to identify possible specific covalently bound fragment compounds. We report on the successful implementation of this strategy when applied to the discovery of novel covalent chemotypes targeting the anti-apoptotic protein hMcl-1.

## Results

### Electrophile-first screening strategy

**Figure 1.**
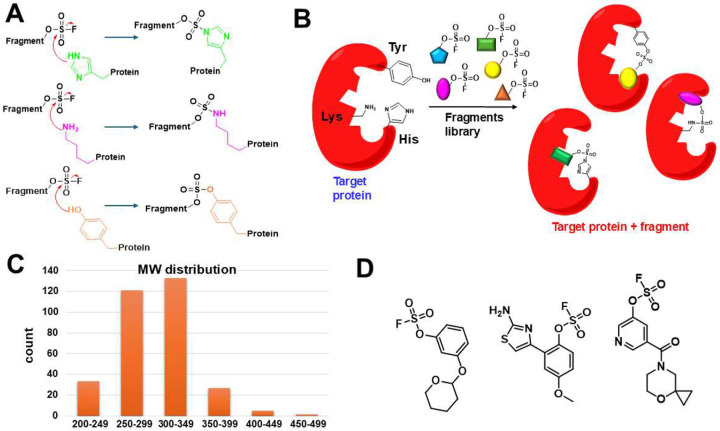
Electrophile-first approach for covalent targeting of Lys, His, or Tyr residues. **A**) The covalent bond formation between protein targets and aryl-fluorosulfates can lead to stable adducts including imidazole sulfamates (for His), amino sulfamates (for Lys), or sulfates (for Tyr). **B**) Schematic representation of the fragment library deployed to target His, Lys, or Tyr residues. **C**) MW distribution of the 320 fragments library. **D**) Chemical structures of representative compounds from the library. A complete list is provided as supplementary **Table S1**.

To assess if an electrophile-first approach can be successful for the unbiased, *de novo* identification of covalent fragments targeting His, Lys, or Tyr residues we assembled an initial compound library of 320 aryl-fluorosulfates with fragment-like properties (Figure 1, supplementary **Figure S1,** supplementary **Table S1**). While the synthesis of these agents can be relatively easily accomplished by the corresponding phenolic fragment and proper catalyst reagents^43^, we opted to assemble the library from commercially available compounds at first (Enamine, supplementary **Table S1)**. Ideal fragment hits are expected to form stable and specific 1:1 complex with any given His residue, forming an irreversible imidazole sulfamate, and/or with Lys, forming stable and irreversible sulfamate, and/or with Tyr, forming a stable sulfate (Figure 1).

**Figure 2.**
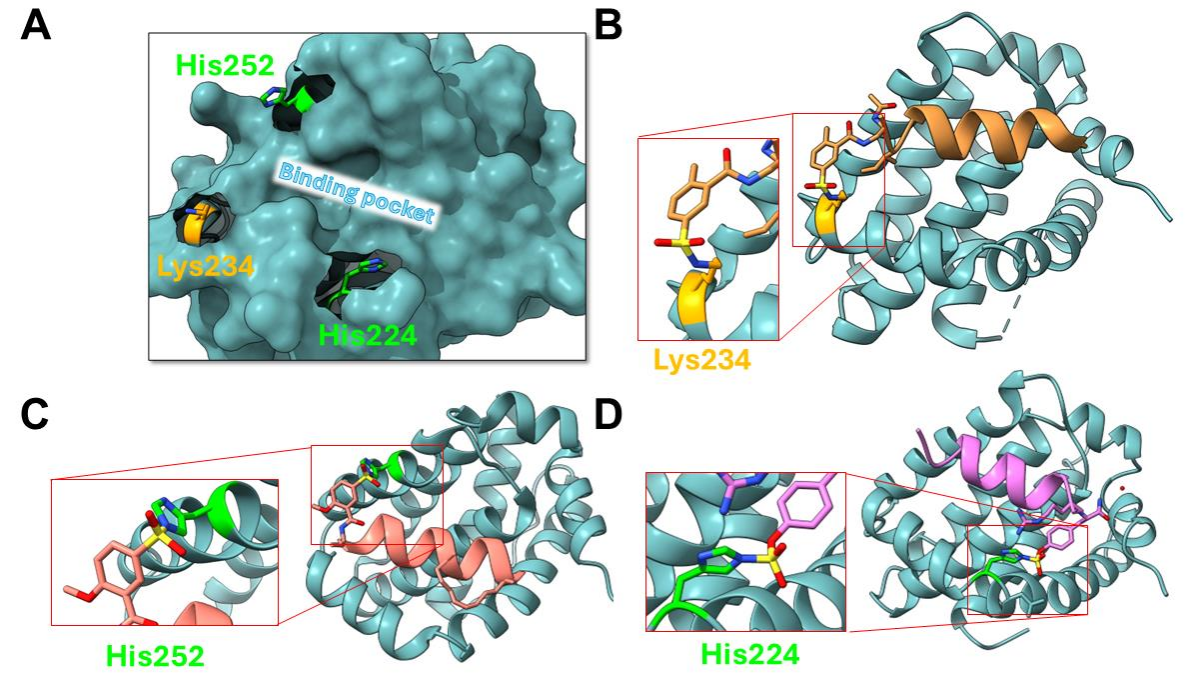
Lys- and His-covalent BH3-based antagonists derived recently against hMcl-1. **A**) Surface representation of hMcl-1(172–323) highlighting the BH3 binding pocket and the binding site Lys 234, His 224, and His 252 residues. **B**) Ribbon representation of the crystal structure of the first covalent BH3-peptide in complex with hMcl-1 targeting Lys 234 using a sulfonyl fluoride. Peptide’s sequence is Ac-Dap(2Me,5FSB)IAEQLRRIGDRF-CONH_2_ where 2Me,5FSB = 2-methyl, 5-sulfonyl fluoride (PDB ID 6VBX)^26^. **C**) Ribbon representation of the crystal structure of the first His 252 covalent stapled BH3 peptide in complex with hMcl-1. The peptide has sequence Ac-Dap(2MeO,5FSB)IAEQLRXIGDXF-CONH_2_, where X indicates the hydrocarbon staple formed from two (S)-2-(4-pentenyl)Ala (metathesis reaction) (PDB ID 8VJP)^22^. **D**) Ribbon representation of the crystal structure of the first His 224 covalent BH3-peptide (named peptide **6**) in complex with hMcl-1 (PDB ID 9CKN)^42^. Peptide **6** sequence is Ac-IAEQLRRIGDRZ-CONH_2_ where Z is a fluorosulfate ((S)-2-amino-3-(4-((fluorosulfonyl)oxy)phenyl) propanoic acid).

**Figure 3.**
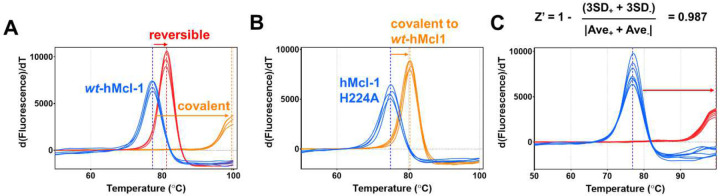
Denaturation thermal shift curves for hMcl-1(172–323) measured in absence and presence of various ligands. **A**) ΔTm curves for *wt*-hMcl-1(172–323) measured in absence (blue) and presence of a reversive binding peptide (red, peptide **7** of sequence Ac-IAEQLRRIGDRY-CONH_2_) and its equivalent peptide **6** that targets hMcl-1(172–323) covalently via a fluorosulfate targeting His 224 (Figure 2). **B**) As in **A**) but the covalent peptide is tested against a hMcl-1(172–323) His224Ala mutant. **C**) Determination of Z’ factor based on repeated measurements for hMcl-1(172–323) in absence (blue) and presence (red) of a reference Bim BH3 peptide.

As a model system to define the most suitable screening approach with our library, we chose the anti-apoptotic protein hMcl-1(172–323), given that we recently reported on ligand-first strategies to derive covalent peptide inhibitors targeting active site residues Lys 234^26^, or His 252^22^ with more reactive sulfonyl fluorides, or more recently also His 224 with an aryl-fluorosulfate peptide^42^ (Figure 2). Based on our previous experience with covalent agents, we observed that covalent compounds could cause very large denaturation thermal shifts, often with double-digit degrees Celsius, compared to reversible agents. For example, the denaturation thermal shift for hMcl-1(172–323) is ~ 78 °C, while the binding to His 224 by covalent peptide **6** (Figure 2) caused a shift of 22.04 +/− 0.01 °C degrees^42^, which is much larger than the same compound lacking the fluorosulfate (peptide **7**, ΔTm = 3.9 +/− 0.01 °C (Figure 3A). Mutation of the targeted His 224 resulted in a ΔTm value for this mutant induced by compound **6** that is much smaller (5.50 +/− 0.05 °C) compared to the effect on the wild-type protein (Figure 3B).

Hence, to assess if ΔTm measurements could be reliably deployed for higher throughput of covalent fragment library screening, we measured the reproducibility of this assay, which resulted in a Z’ factor of 0.987 (Figure 3C).

Hence, ΔTm measurements represent in principle a simple and cost-effective screening strategy that could be used to screen for possible covalent agents in the fragment library. However, much like in any fragment screening campaign, we didn’t expect the compound library to contain very potent agents at first. Hence, to shift the equilibrium among possible binders toward the formation of the covalent adduct, we increased not only the compound/protein ratio, as typical in fragment screening (>10:1), but also the incubation time (we used up to 48 h) given the irreversible nature of the approach. The latter strategy was possible because of the excellent aqueous stability of the aryl-fluorosulfates (supplementary **Figure S2**)^23,42^. Using this simple strategy, each compound was assessed for its effect on the denaturation temperature of hMcl-1(172–323), and primary positive hits are considered those inducing a significant change in thermal denaturation (ΔTm of greater than +/− 1.2 °C; supplementary **Figure S3**). Using those criteria, a total of 11 primary hits were selected, corresponding to 3 inducing a positive ΔTm and 8 inducing a negative ΔTm (supplementary **Table S2**). Those fragments hits were further investigated for their ability to form a stable 1:1 adduct with hMcl-1(172–323) via mass spectrometry analysis under the same experimental conditions used for the thermal shift measurements (supplementary **Table S2**). Of the 11 hits, 5 did not form a covalent adduct with the protein hence were no further considered. To assess if the 6 remaining hits covalently bound to any of the binding site residues (Figure 2), three single-point mutants were prepared with either Lys 234, His 252, or His 224 mutated to Ala, and ΔTm values for these mutants were measured in the presence of the hit compounds (supplementary **Table S2**). A}we found that among those 6 primary hit fragments, ΔTm values induced by 2 hit compounds were reduced when tested against hMcl-1(172–323) His224Ala mutant (Table 1, supplementary **Figure S4**), suggesting that those 2 hits bound to His 224. Further validation was obtained by the fact that while we detected the formation of a 1:1 complex using mass spectrometry with *wt*-hMcl1(172–323), only a modest amount of covalent adduct was observed with the His224Ala mutant (Table 1; supplementary **Figure S5**). Not surprisingly, with fragment **2**, which induces the largest ΔTm, hence likely the most effective under the chosen experimental conditions, we observed nearly 100% 1:1 covalent complex formation via MS analysis (Table 1; supplementary **Figure S5**). These data establish that two structurally related novel hit compounds (Table 1) could be identified targeting covalently hMcl-1(172–323) His 224. Hence in this screening campaign aimed at discovering binding site covalent hits, 2 hits inducing a positive thermal denaturation stabilization were identified. While in this example only compounds causing a positive shift were identified and considered for further studies, agents that cause negative shifts could also be of interest, if specific^44^.

### Structural characterization of hit 2 and structure activity relationship studies

**Figure 4.**
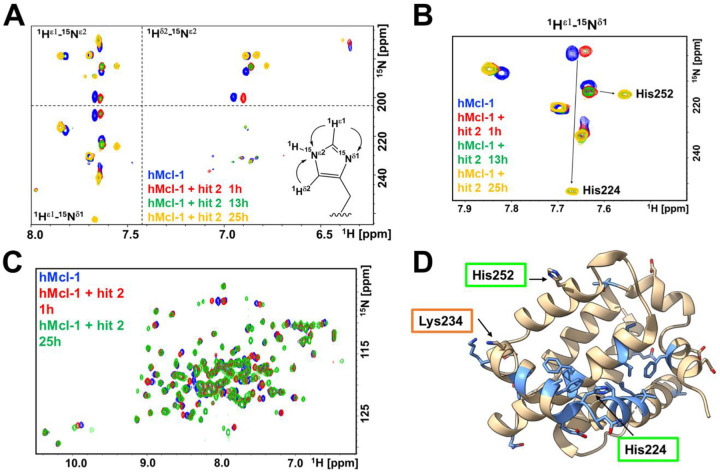
Heteronuclear [^15^N,^1^H] NMR correlation spectra and chemical shift mapping using ^15^N labeled hMcl-1(172–323) measured in absence and presence of fragment hit **2** at various incubation times. **A**) Overlay of 2D long-range [^15^N,^1^H] correlation spectra for His side chains collected in absence (blue) or presence of fragment hit **2** after various incubation times (red, 1 h; green, 13 h; yellow, 25 h). **B**) Enlarged region of the spectra reported in **A**) highlighting the time-dependent large chemical shift perturbations of His 224 side chain resonances. Resonance assignments were obtained as we reported recently^22,42^ and further confirmed by single-point mutations (supplementary **Figure S6**). **C**) Backbone 2D [^15^N,^1^H] correlation spectra for ^15^N-hMcl-1(172–323) measured in absence (blue) or presence of fragment hit **2** after various incubation times (red, 1 h; green, 25 h). Resonance assignments are reported in supplementary **Figure S7**. **D**) Mapping the observed backbone chemical shift perturbations into the 3D structure of hMcl-1(172–323) (blue) reveals that the compound is likely bound in proximity to His 224 in the BH3 binding pocket.

To study the interactions between the fragment hit and hMcl-1(172–323) at the atomic level we deployed solution NMR spectroscopy studies with a sample uniformly ^15^N labeled of hMcl-1(172–323) and X-ray crystallography. First, to ascertain if the hit interacted covalently with His 224, we measured 2D long range [^15^N, ^1^H] correlation spectra for His side chain with a ^15^N-labeled sample of hMcl-1(172–323), collected in absence and in presence of the fragment hit **2** (Figure 4; supplementary **Figure S6**). The spectral data clearly indicated a large perturbation to the ^15^N^ε^ and ^15^N^δ^ resonances of His 224, compatible with a covalent bond formation at the imidazole ring of His 224. Chemical shift perturbations studies mapping the changes in backbone [^15^N, ^1^H] correlations of hMcl-1(172–323) in presence of fragment hit **2**, also revealed that the compound likely binds to the BH3 binding region, closer to His 224 than other binding site nucleophilic residues His 252, or Lys 234 (Figure 4; supplementary **Figure S6**).

**Figure 5.**
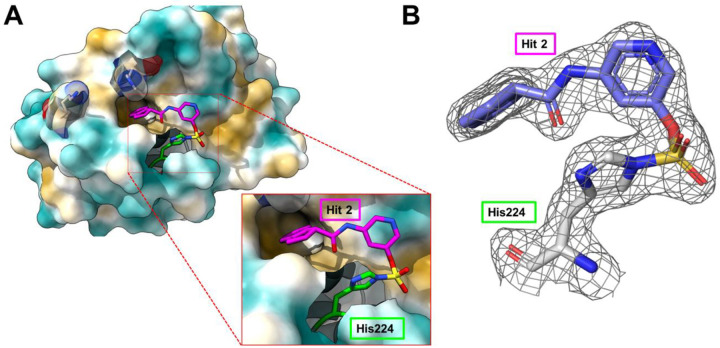
Crystal structure of hMcl-1(172–323) in complex with fragment hit **2** solved at 1.82 Å resolution. **A**) Surface representation of hMcl-1(172–323) highlighting the position of fragment hit **2** covalently bound to the side chain of His 224. The other binding site nucleophilic residues His 252 and Lys 234 are also highlighted. The insert displays a close-up view of the imidazole sulfamate bond resulting from the reaction of the fluorosulfate of fragment hit **2** and the side chain of His 224. **B**) The 2Fo-Fc map of the bound fragment hit **2** contoured at 1.0 sigma, highlighting the contiguous electron density map between fragment hit **2** and the protein (PDB ID 9EFJ).

Finally, the X-ray structure of the complex between fragment hit **2** and hMcl-1(172–323) was also obtained at 1.82 Å resolution, indicating that the compound binds in a deep region within the BH3 binding pocket surrounding His 224 (Figure 5A). In addition, the contiguous electron density between the compound and His 224 N^ε^ that corresponds to the expected imidazole sulfamate moiety can be clearly observed (Figure 5B). Analysis of the structure of the complex suggests several possible routes of optimizations, including for example introducing more hydrophobic substituents on the indane ring.

**Figure 6.**
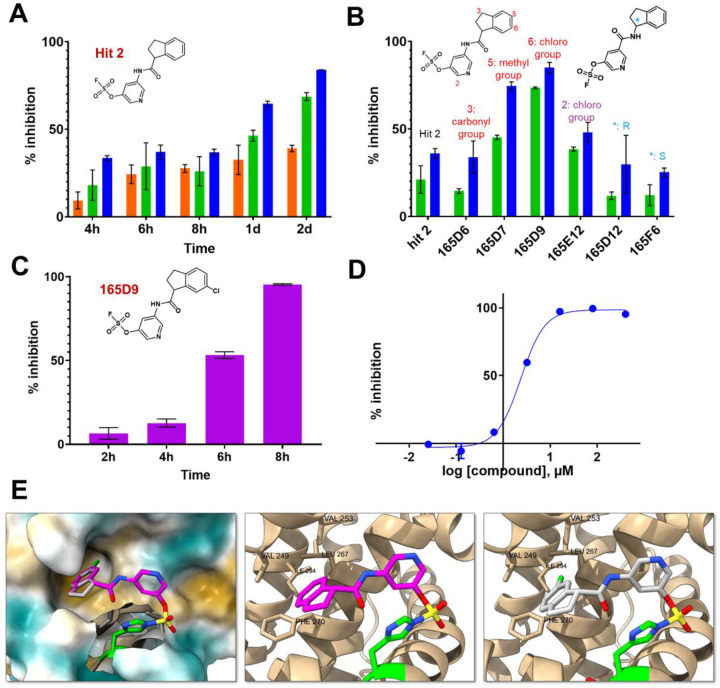
Structure-activity relationships studies and inhibition properties of fragment hit **2** and analogs as determined by a DELFIA displacement assay. **A**) Percent inhibition in a DELFIA displacement assay with fragment hit **2** measured at various incubation times and different concentrations (10 μM, orange; 50 μM, green; 100 μM, blue). **B**) Percent inhibition in the DELFIA displacement assay for the synthesized compounds (chemical structures are shown) as determined at two concentrations (50 μM, green; 100 μM, blue) after 4 h incubation time. **C**) Percent inhibition in a DELFIA displacement assay with compound **165D9** (its chemical structure is shown) measured at various incubation times at 15 μM. **D**) Dose response displacement assay with compound **165D9** (24 h incubation time) leading to an IC_50_ value of ~ 2.5 μM (supplementary **Figure S9**). **E**) Comparison of the structures of hMcl-1(172–323) in complex with his **2** (magenta) (PDB ID 9EFJ) and **165D9** (gray). The model of the complex with **165D9** was built using Sybyl-X based on the X-ray coordinates of fragment hit **2** in complex with hMcl-1(172–323) (PDB ID 9EFJ). The Cl atom in position 6 of the indane ring nicely occupies a deep hydrophobic pocket where the ligand binds. The hydrophobic residues are also highlighted surrounding the Cl atom are highlighted.

Preliminarily, the re-synthesis of fragment hit **2** and a few analogs was accomplished by coupling proper 1-carboxy-indane with various hydroxy-anilines, that are subsequently converted in the respective fluorosulfates (supplementary **Figure S8**)^43^. An alternative structure where the amide bond was inverted was also obtained (Figure 6; supplementary **Figure S8**). Biochemical assays to measure the ability of the agents to displace the binding between hMcl-1(172–323) and a biotinylated-BH3 peptide can be used to determine IC_50_ values at a given incubation time, via a DELFIA assay platform as we described previously^22,26,42^. Hence, rank ordering of fragment hit **2** and its analogs could be accomplished by measuring their ability to prevent the binding of a reference biotinylated-BH3 BIM peptide in the DELFA assay at a given incubation time (Figure 6) as we reported previously^22,26,42^. Time and concentration dependent inhibition was observed as expected by the nature of the covalent hit (Figure 6A). When tested at 50 μM, fragment hit **2** was able to inhibit about 50% of the binding between hMcl-1(172–323) and the reference BH3 peptide (Figure 6A) after 24 h incubation. The same assays were used to rank order the analogs in our preliminary SAR studies (Figure 6B). Based on these data we observed that inverting the amide bond did not result in an improved affinity for hMcl-1(172–323), while increasing the hydrophobic nature of the substituents in the indane ring resulted in compounds with increased affinity (Figure 6B). Among those, compound **165D9**, presenting a -Cl in position 6 of the indane ring, displayed the largest inhibition in the DELFIA assay (Figure 6B). The time dependent percent inhibition for this compound increased significantly, with nearly complete inhibition already at 15 μM and with only 8 h incubation time (Figure 6C). A dose-response DELFIA curve resulted in an IC_50_ value in low micromolar range after 24 h incubation (Figure 6D; supplementary **Figure S9)**

**Figure 7.**
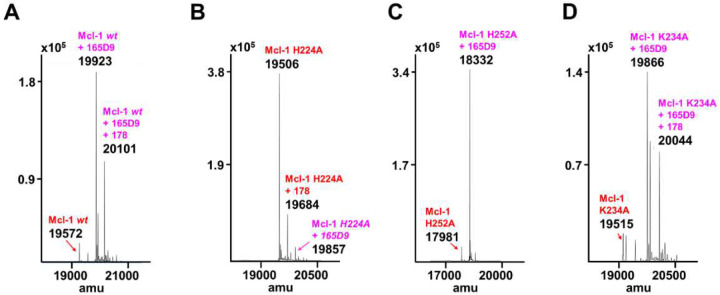
Mass spectrometry analyses of the optimized hit **165D9** when tested against hMcl-1(172–232) and its mutants. The mass spectrometry data for panels **A-D** are tabulated also in supplementary **Table S3.** In some *E.coli* expressed hMcl-1(172–323) constructs we had previously observed a +178 that corresponds to the mass after phosphogluconosylation of the target^22,42^.

Agent **165D9** was also characterized via ΔTm measurements and mass spectrometry analyses against *wt*-hMcl-1(172–323) and the three mutants as described in Table 2. Remarkably, agent **165D9** after only 8 h incubation induced a denaturation thermal shift stabilization of ~10 °C, compared to ~ 4 °C as observed for fragment hit **2** after 48 h incubation (Table 2, supplementary **Figure S10**). Under the same experimental conditions, a 1:1 covalent adduct was observed via MS analyses with *wt*-hMcl-1, hMcl-1 His252Ala, and hMcl-1 Lys234Ala, but not with hMcl-1 His224Ala mutant (Table 2; Figure 7), clearly suggesting the specific covalent interaction with His 224.

Hence, following the primary and secondary screening, and the identification of two related hit compounds, a limited SAR study resulted in **165D9** that represents a novel His 224 covalent chemotype, with a relatively high affinity for hMcl-1, and that is readily amenable to further hit to lead optimizations based on the obtained structural characterizations of the complex.

Finally, to anticipate whether the ligands would also bind covalently in the more acidic tumor environment, we measured long-range [^15^N, ^1^H] correlation spectra for His side chains at various pH values (supplementary **Figure S11**). Even at pH 6.5, the chemical shift pattern of His 224 remains in the unprotonated configuration, and upon exposure of the sample with fragment hit **2**, large perturbations of its resonances can be observed, indicative of covalent binding (supplementary **Figure S11**).

## Discussion and conclusions

Covalent drugs provide significant pharmacodynamic and pharmacokinetic advantages over reversible agents. In oncology, these efforts resulted in several new approved drugs in recent years. However, covalent strategies have been developed only to target cysteine (Cys) residues, which are rarely found in targets’ binding sites. Among other nucleophilic residues that could in principle be targeted for the design of covalent drugs with proper electrophiles are the side chains of histidine (His), lysine (Lys), or tyrosine (Tyr). Aryl-fluorosulfates are mild electrophiles that are very stable in aqueous buffer, in biological media^29^, and even in vivo^23^. We and others demonstrated that despite their mild nature, aryl-fluorosulfates can efficiently react with the side chains of Lys, Tyr, or His residues, when properly juxtaposed to the targeted residues by a high affinity ligand^23,29,30,42^. However, a fragment-based electrophile-first approach may be more powerful for the *de novo* identification of covalent ligands, possibly unraveling suitable nucleophilic residues and/or perhaps novel allosteric pockets that can be covalently targeted. While this strategy has been proven for targeting Cys residues, a similar approach with aryl-fluorosulfates has not yet been reported. Here, we demonstrated that the use of denaturation thermal shift analysis is potentially a powerful and cost-effective strategy to screen a fluorosulfates fragment library against a challenging drug target such as hMcl-1. Our approach is based on the large denaturation thermal shifts induced by covalent ligands, and on shifting the equilibrium of the weakly interacting fragments by increasing not only the ligand-protein ratio but also the incubation time. The latter is possible thanks to the high aqueous stability of fluorosulfates, while the same strategy is generally not possible with the more reactive sulfonyl fluorides, that have a more limited aqueous stability^27^. The identification of the targeted residues is also easily accomplished by a variety of approaches, including measurements of ΔTm and mass analyses with mutant constructs (Tables 1 and 2), or detection of chemical shift perturbations in long-range heteronuclear NMR measurements with ^15^N labeled protein, for His targeting fragments (Figure 4). It is interesting that out of 320 fragments tested, only one residue, namely His 224, emerged as the most suitable target. This is not entirely surprising based on our recent evaluations of His reactivity using a ligand-first approach against the same drug target, in which we identified that His was preferred to Lys for targeting by both sulfonyl fluorides and fluorosulfates, and that His 224 is more reactive to fluorosulfates than His 252^22,42^. Hence, these results once again underline the suitability of His side chains to a covalent targeting approach, given that His is in theory the second most nucleophilic residue after Cys, that its side chain is considerably more rigid than Lys, and that His residues are also often found in protein binding sites.

Our screening approach resulted in the initial fragment hit **2** whose binding geometry as determined by X-ray crystallography is novel with respect to the several hMcl-1 antagonists reported to date, that almost invariably anchor around an electrostatic interaction with Arg 263 and an acid residue (mimicking a critical Asp acid in the BH3 peptides). Limited structure-activity relationships led to compound **165D9** with an IC_50_ value of ~2.5 μM in a DELFIA displacement assay (Figure 6). Hence, this relatively simple screening strategy led to novel information on the design of possible future hMcl-1 targeting covalent therapeutics that structurally dramatically depart from current hMcl-1 antagonists.

In conclusion, our studies report for the first time an electrophile-first strategy to derive covalent agents with a small library of aryl-fluorosulfates and the associated biophysical strategies to screen and characterize hits that are of general applicability and could open the way to entirely new classes of covalent targeted therapeutics.

## Methods

### General materials and instrumentation.

All reagents used were commercially available. For the synthesis of the reported analogs, we followed the synthetic scheme reported in supplementary **Figure S8**. Each intermediate and final product was purified using a preparative RP-HPLC using an XTerra C18 column (Waters) with a JASCO preparative HPLC system. The gradient used in the purification is water/acetonitrile (5% to 100%) containing 0.1% TFA (purity > 95%). The identity of the compounds was confirmed by high-resolution mass spectrometry.

### Covalent fragment library preparation.

The library utilized for the initial screening consisted of 320 commercially available aryl-fluorosulfates. The individual compounds were dissolved in deuterated DMSO to achieve a concentration of approximately 25 mM.

### Mass spectrometry.

The masses of *wt*-hMcl-1(172–323) and the reported mutants, analyzed in absence and presence of the fragment hits, were performed using an Agilent 6545 QTOF LC/MS instrument (Table 1, Table 2, Figure 7, supplementary **Figure S5,** and supplementary **Table S3**).

### NMR spectroscopy.

Protein NMR spectra were acquired on a Bruker Avance III 700 MHz spectrometer equipped with a TCI cryoprobe. The stability of several aryl-fluorosulfates was tested with a 1D ^1^H NMR experiment, analyzing 100 μM of the fragment hits in 50 mM phosphate buffer pH = 7.5, 150 mM NaCl, 1 mM DTT, 10% D_2_O, 1% D6 DMSO, at T = 25 °C, at different time points (0 min, 1 d, and 2 d incubation). For the detection of His side chain resonances, 2D-[^15^N,^1^H]-long range so fast HMQC spectra were optimized to detect the imidazole ring ^2^*J* ^15^N-^1^H correlations with a uniformly ^15^N-labeled sample (50 μM) of *wt*-hMcl-1(172–323) or of the mutants. Resonance assignments for the His side chains were obtained as we reported previously^22,42^. For the 2D [^15^N,^1^H] HSQC experiment, 50 μM of hMcl-1(172–323) was incubated with and without 1 mM of the fragment hit **2** and recorded at different time points. Data processing was obtained using TopSpin 4.4.1 (Bruker, Billerica, MA).

### Denaturation thermal shift assays.

Denaturation thermal shift assay measurements were performed using QuantStudio 3 (Thermo Fisher Scientific), and the results were analyzed with Protein Thermal Shift Software 1.3. For the library screening and the fragment hits confirmations, the samples were prepared by incubating 10 μM of protein (*wt*-hMcl-1(172–323), hMcl-1(172–323) Lys234Ala, hMcl-1(172–323) His252Ala, or hMcl-1(172–323) His224Ala) with ~500 μM of the fragment hits at various incubation times. Protein and hits were dissolved in 50 mM phosphate buffer, pH 7.5, 150 mM NaCl, 1 mM DTT, 10X Sypro orange, 2% DMSO. The samples were heated from 10 °C to 95 °C. For the Z’ factor calculation, the temperature increased up to 99.5 °C. A linear temperature increase of 2 °C/min was used and the fluorescence intensity was measured with Ex/Em: 550/586 nm.

### Biochemical assay.

An heterogeneous assay based on the DELFIA (Dissociation-enhanced lanthanide fluorescent immunoassay) platform was developed for 6His-tagged-hMcl-1(172–323) sample (16 nM), as previously described^26^. Specifically, we prepared a solution containing a reference biotinylated-BH3 peptide at 600 ng/ml (the exact composition of the peptide is biotin-aminohexanoic acid-IWIAQELRRIGDEFNAYYARR-CONH_2_). 100 μL of the solution was added to each well of the streptavidin-coated plates (96 well, PerkinElmer) for 2-h, and subsequently the plates were washed (3 times). A solution containing the target protein expressed with a 6His-tagged-hMcl-1(172–323) and the fragment hits were pre-incubated at various times ans concentrations, and then added to BH3-peptide bound streptavidin-coated wells. At this point a specific Europium-tagged anti-6xHis antibody (PerkinElmer, 1:2000) solution was added and further incubated for 2 h on a microplate shaker. Plates were then subsequently washed 3 times. Finally, prior to reading with a VICTOR X5 microplate reader (excitation and emission wavelengths of 340 and 615 nm), each well was incubated with 200 μL of the enhancement solution (PerkinElmer) for 10 min. Prism 10 (GraphPad) was used to analyze the data. Dose-response inhibition curves (Figure 6D; supplementary **Figure S9**) were obtained at various protein-ligand pre-incubation times as reported (4 h or 24 h) and data were analyzed and plotted using Prism 10 (GraphPad). The rank ordering of fragment hit **2** and its analogs was conducted by incubating 50 μM and 100 μM of fragments for 4 h and by measuring their ability to prevent the binding of the reference biotinylated-BH3 peptide to hMcl-1(172–323) (Figure 6B).

### Protein expression and purification.

Protein expression and purification were accomplished as we recently described to obtain the ligand-binding domain of *wt*-hMcl-1(172−323) and of the mutants, either in unlabeled or uniformly ^15^N-labeled form^22,42^. All protein samples were expressed with an N-terminal His tag and a thrombin cleavage site, to simplify the purification process. The proteins were purified using immobilized metal ion affinity chromatography (IMAC) with a linear gradient of imidazole (elution buffer: 25 mM Tris at pH 7.5, 500 mM NaCl, 1 mM DTT, and 500 mM imidazole), and further purified through size-exclusion chromatography with a HiLoad 26/60 Superdex 75 preparative-grade column. The protein samples used for all the experiments were prepared in aqueous buffer composed of 50 mM phosphate at pH 7.5, 150 mM NaCl, and 1 mM DTT. In the ^15^N labeled proteins used for the detection of His side chain resonances, the 6 x histidine tag was removed during the purification process.

### X-ray crystallography.

Crystallization was conducted using sitting drop vapor diffusion at 4°C, with diffraction quality crystals grown in 0.15 M Potassium Bromide and 30 % (w/v) PEG 2000 MME. The 15.3 mg/mL MCL-1/E4P5 complex was plated at 0.375 μL:0.375 μL ratio (protein:mother liquor) using SPT Labtech Mosquito LCP. Crystals were cryo-protected in 20% Ethylene glycol and flash frozen in liquid nitrogen. Data collection was conducted at the Diamond Light Source (I04) beamline. A data set was collected on a crystal that diffracted to 1.8Å, and the diffraction data was processed in the P1211 space group using XIA2 Dials. Molecular replacement for the data set was performed using a search model based on PDB ID:6P3P in PHASER, PHENIX. The top solution was refined using restrained refinement in REFMAC, CCP4. Several rounds of refinement and model building were performed in the absence of ligand using COOT, CCP4 and PHENIX. After placement of the solvent molecules, the chemical model of the ligand, fragment hit **2**, was fit into the remaining density and refined by REFMAC, CCP4. The final crystal data statistics are listed in supplementary **Table S4**. The coordinates for the complex between fragment hit **2** and hMcl-1 have been deposited in the PDB and will be released upon publication (PDB ID 9EFJ).

## Figures and Tables

**Table 1.** Chemical structures of positive covalent hits and induced ΔTm values against *wt*-hMcl-1(172–323) and mutants as indicated (supplementary **Figure S4**). The percentage of adduct formation as measured by mass spectrometry analyses against *wt*-hMcl-1(172–323) and the mutants is also reported (supplementary **Figure S5**).

		ΔTm (°C) and % complex formation (MS)			
Structure	MW	hMcl-1 *wt*	hMcl-1 H224A	hMcl-1 H252A	hMcl-1 K234A
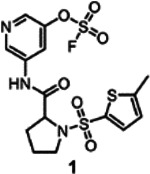	449.5	**2.11 ± 0.01**	−0.34 ± 0.09	2.90 ± 0.17	1.96 ± 0.17
		**~ 55%**	< 5%	~ 60%	~ 60%
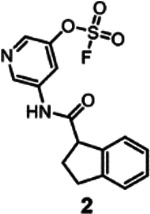	336.3	**3.97 ± 0.17**	−1.28 ± 0.17	6.57 ± 0.26	4.77 ± 0.26
		**~ 95%**	< 25%	~ 98%	~ 95%

**Table 2.** Chemical structure of the optimized analog **165D9,** and ΔTm values and mass spectrometry analyses against *wt*-hMcl-1(172–323) and its mutants as indicated (supplementary **Figure S10**).

		ΔTm (°C) and % complex formation (MS)			
Structure	MW	hMcl-1 *wt*	hMcl-1 H224A	hMcl-1 H252A	hMcl-1 K234A
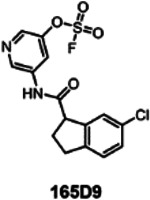	370.02	**10.23 ± 0.05**	−1.15 ± 0.04	10.44 ± 0.06	9.96 ± 0.20
		**~ 98%**	< 2%	~ 98%	~ 98%

## Data Availability

The atomic coordinates of the model between hMcl-1(172–323) and the fragment hit **2** have been submitted to the protein data bank (PDB ID Code 9EFJ). Authors will release the atomic coordinates and experimental data upon article publication.

## Abbreviations used:

DELFIA: Dissociation-Enhanced Lanthanide Fluorescent Immunoassay
HMQC: Heteronuclear Multiple Quantum Coherence
HPLC: High-performance liquid chromatography
